# *TP53* somatic mutations are associated with poor survival in non-small cell lung cancer patients who undergo immunotherapy

**DOI:** 10.18632/aging.103502

**Published:** 2020-07-22

**Authors:** Liqin Zhao, Xiaofei Qu, Zhenhua Wu, Yuehua Li, Xiaowei Zhang, WeiJian Guo

**Affiliations:** 1 Department of Medical Oncology, Fudan University Shanghai Cancer Center, Shanghai, China; 2 Department of Oncology, Shanghai Medical College, Fudan University, Shanghai, China; 3 Cancer Institute, Fudan University Shanghai Cancer Center, Shanghai, China; 4 Department of Medical Oncology, The First Affiliated Hospital of University of South China, Hunan, China

**Keywords:** TP53, somatic mutation, tumor mutation burden, immunotherapy, non-small cell lung cancer

## Abstract

In this study, we investigated the association between *TP53* somatic mutations and immunotherapeutic outcomes in non-small cell lung cancer (NSCLC) patients. Kaplan-Meier survival curve analysis of the MSK-IMPACT cohort of 350 NSCLC patients shows that overall survival (OS) is significantly lower for patients with truncating *TP53* mutations than those with wild-type *TP53* (OS: 9 months vs. 14 months; *P*=0.019). Multivariate analysis shows that truncating *TP53* mutations are an independent predictor of immunotherapeutic outcomes. Moreover, among NSCLC patients with lower tumor mutation burden (TMB), those with *TP53* truncating mutations showed significantly lower OS than those with wild-type *TP53* [hazard ratio (HR) = 1.40, confidence interval (CI) = 1.13-1.73; *P* = 0.002]. *TP53* mutations correlate with higher infiltration of CD8^+^ T cells, neutrophils and dendritic cells in lung adenocarcinoma tissues. A prognostic model with *TP53* mutational status shows better survival prediction than the model without *TP53* mutational status 1-year [area under curve (AUC): 64.9% vs. 60.2%; *P* = 0.052] and 2-years (AUC: 70.9% vs. 66.1%; *P* = 0.098) post-immunotherapy. These findings demonstrate that truncating *TP53* mutations correlate with poor immunotherapy outcomes in NSCLC patients with low TMB. *TP53* mutation status also improves the prognostic prediction in NSCLC patients that underwent immunotherapy.

## INTRODUCTION

Lung cancer is one of the leading causes of cancer-related deaths worldwide; non-small cell lung cancer or NSCLC accounts for over 85% of lung cancer cases [1, 2]. The long term outcomes for metastatic NSCLC patients are poor despite significant therapeutic advances including the availability of small molecule inhibitors against epidermal growth factor receptor tyrosine kinases (EGFR-TK), anaplastic lymphoma kinase (ALK) and c-ros oncogene 1 receptor tyrosine kinase (ROS1) because majority of patients report drug resistance [3, 4]. The emergence of immune checkpoint inhibitors (ICI), which includes targeted antibodies against programmed cell death protein 1 (PD-1), programmed cell death-ligand 1 (PD-L1) and cytotoxic T-lymphocyte-associated protein 4 (CTLA4) has increased the survival of metastatic NSCLC patients, but, only a small number of metastatic patients achieve long-term survival [5].

PD-L1 expression and tumor mutation burden (TMB) have been used as clinical biomarkers to predict the response to PD-1/PD-L1 antibody therapy [5, 6], but, the prediction efficacy is sub-optimal when the PD-L1 expression is less than 50% in EGFR^+^/ALK^+^ NSCLC patients and non-squamous lung cancer patients [7]. Moreover, there is no consensus regarding the use of TMB as a selection criteria for PD-1/PD-L1 antibody therapy because it would require whole exome sequencing or large scale sequencing of several target genes in the tumor samples of NSCLC patients [6, 8]. A recent study showed that mutations in *POLE* and *POLD1* are potential biomarkers that can effectively predict treatment outcomes of immunotherapies in several cancers, including lung cancer [9].

Several studies suggest that *TP53* gene mutations are potential prognostic biomarkers for cancer patients that undergo immunotherapies, such as head and neck squamous cell cancer, lung adenocarcinoma [10, 11]. However, the association between *TP53* mutations and the efficacy of immunotherapy remains ambiguous. Therefore, in this study, we investigated the association between *TP53* mutations and immunotherapy outcomes of NSCLC patients in a cohort of 350 metastatic or unresectable NSCLC patients who were treated with immunotherapies. This cohort included patients with *TP53* non-truncating mutations as well as those with putative truncating mutations because of frameshift, nonsense or splice-site mutations that reduce TP53 protein expression and function [12–14]. We also tested the prognostic prediction efficacy of a survival model that includes *TP53* mutation status as a parameter.

## RESULTS

### NSCLC patients with *TP53* truncating mutations in the MSK-IMPACT show lower overall survival than those with wild-type *TP53*

**Figure 1:**
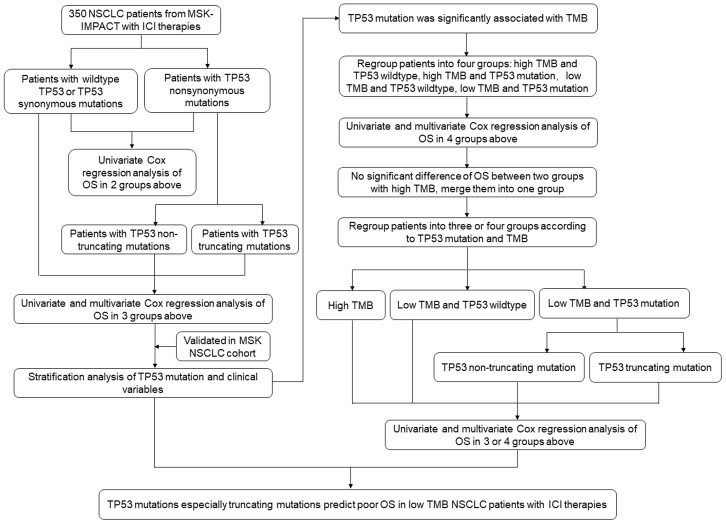
**Flowchart of the study strategy.**

**Figure 2:**
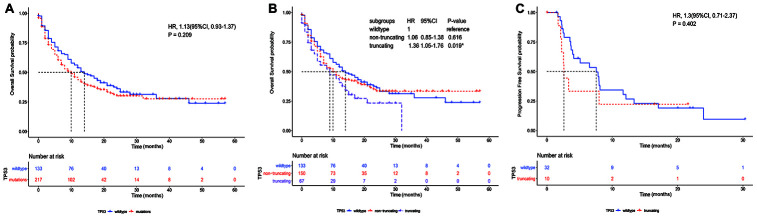
**The association between *TP53* mutation status and overall survival of NSCLC patients treated with immunotherapies.** (**A**) Kaplan-Meier survival curve analysis shows overall survival of NSCLC patients with *TP53* mutations (n=217) and wild-type *TP53* (n=133) in the MSK-IMPACT NSCLC cohort. (**B**) Kaplan-Meier survival curve analysis shows the overall survival of NSCLC patients with wild-type *TP53* (n=133), *TP53* non-truncating mutations (n=150) and *TP53* truncating mutation*s* (n=67) in the MSK-IMPACT NSCLC cohort. (**C**) Kaplan-Meier survival curve analysis shows the progression free survival (PFS) of patients with *TP53* truncating mutations (n=10) and wildtype *TP53* (n=32) in the MSK-NSCLC cohort.

The flow chart of the study strategy is shown in Figure 1. The clinicopathological characteristics of the 350 metastatic or unresectable NSCLC patients from the Memorial Sloan Kettering Cancer Center Integrated Mutation Profiling of Actionable Cancer Targets (MSK-IMPACT) cohort who received anti PD-1/PD-L1 monotherapy (n=329) or a combination of anti-CTLA-4 and anti-PD-1/PD-L1 immunotherapies (n=21) is shown in Table 1. Kaplan-Meier survival curve analysis showed that the median overall survival (OS) of NSCLC patients with *TP53* mutations (n=217) was 10 months compared to 14 months for patients with the wildtype *TP53* (n = 133), but, the differences were not statistically significant [hazard ratio (HR) = 1.13, confidence interval (CI) = 0.93-1.37 and *P* = 0.209; Figure 2A]. Furthermore, the median OS of patients with *TP53* truncating mutations (n=67) was significantly lower at 9 months compared to 14 months for patients with wildtype *TP53* (n=133) as shown in Figure 2B (HR = 1.36, CI = 1.05-1.76; *P* = 0.019).

**Table 1:** Clinicopathological characteristics of NSCLC patients in the MSK-IMPACT cohort that underwent ICI treatment.

**Characteristics**	**No. of Cases (%)**
All subjects	350(100)
Age in years	
31-50	34 (9.7)
50-60	75 (21.4)
61-70	119 (34.0)
>71	122 (34.9)
Sex	
Female	180 (51.4)
Male	170 (48.6)
ICI regime	
PD-1/PD-L1	329 (94.0)
Combo	21 (6.0)
Mean of TMB (/Mb)	9.87 (10.03)
Pathological type	
Adenocarcinoma	271 (77.4)
Squamous carcinoma	45 (12.9)
Other NSCLC types	34 (9.7)

MSK-IMPACT: Memorial Sloan Kettering Cancer Center Integrated Mutation Profiling of Actionable Cancer Targets; NSCLC: Non-small cell lung cancer; ICI: immune checkpoint inhibitors; Combo: combination of anti-CTLA-4 and anti-PD-1/PD-L1 therapy; TMB: tumor mutation burden; /Mb: per Mega bases.

### NSCLC patients with *TP53* truncating mutations in the validation cohort show lower OS compared to those with wild-type *TP53*

Kaplan Meier survival curve analysis of the validation cohort of 75 NSCLC patients that underwent immunotherapies (MSK-NSCLC 2018) [15] showed that the median progression free survival (PFS) of NSCLC patients with *TP53* truncating mutations (n=10) was 2.6 months compared to 7.56 months for patients with wild-type *TP53* (n=32), but, the differences were not statistically significant (HR = 1.30, CI = 0.71-2.37, *P* = 0.402; Figure 2C).

### *TP53* truncating mutations are an independent predictor of immunotherapeutic outcomes in NSCLC patients

**Figure 3:**
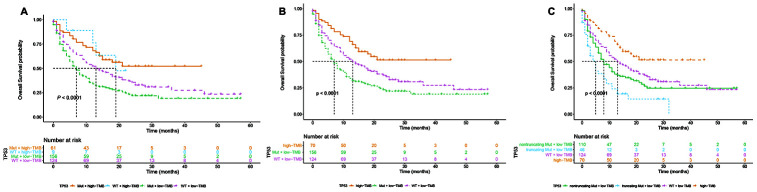
**Stratification subgroup analysis of the relationship between overall survival and *TP53* mutation status in immunotherapy-treated NSCLC patients of the MSK-IMPACT cohort with low or high TMB.** (**A**) Kaplan-Meier survival curve analysis shows the OS of NSCLC patients in the MSK-IMPACT cohort divided into four subgroups, namely *TP53* mutations and high TMB (n=61), *TP53* wildtype and high TMB (n=9), *TP53* mutations and low TMB (n=156), and *TP53* wildtype and low TMB (n=124). (**B**) Kaplan Meier survival curve analysis shows the OS of NSCLC patients with *TP53* mutations and low TMB (median OS=7 months) compared to NSCLC patients with wild-type *TP53* and low TMB (median OS=13 months). (**C**) Kaplan Meier survival curve analysis shows the OS of NSCLC patients with high TMB irrespective of *TP53* mutation, *TP53* truncating mutations plus low TMB, *TP53* non-truncating mutations plus low TMB, and wildtype *TP53* plus low TMB. As shown, NSCLC patients with *TP53* truncating mutations plus low TMB shows the lowest overall survival compared to other groups.

Next, we focused on the correlation between *TP53* truncating mutations and the survival of NSCLC patients undergoing immunotherapies. Supplementary Table 1 shows the 55 truncating mutation sites in the *TP53* gene from these patient samples. Multivariate Cox regression analysis showed that *TP53* truncating mutations were an independent predictor of immunotherapeutic outcomes after the data was adjusted by age, sex, ICI regime and TMB (Table 2). Moreover, the stratification analysis results showed positive correlation between *TP53* mutation status and TMB (*P* <0.001; Table 3). The mean TMB was 5.79, 12.14, and 12.92 for patients with wild-type *TP53*, non-truncating *TP53* mutations, and truncating *TP53* mutations, respectively. While high TMB favors survival [6, 8], *TP53* mutations, especially truncating mutations reduce survival rates. Therefore, analysis of the association between OS and *TP53* mutation status in low or high TMB NSCLC patient subgroups showed that patients with high TMB are associated with longer OS irrespective of the *TP53* mutation status, however, the OS of patients with low TMB was significantly reduced by the *TP53* mutation status (log-rank *P* < 0.0001; Figure 3A). We did not observe any differences in the OS of high TMB patients with or without *TP53* mutation (log-rank *P* = 0.96; Supplementary Figure 1). Therefore, we merged the two subgroups of high TMB patients with or without *TP53* mutations into one group and compared their survival status with the remaining two low TMB subgroups (with and without *TP53* mutations). We observed that patients with low TMB and *TP53* mutations showed significantly shorter median OS of 7 months compared to a median OS of 13 months for those with low TMB and wild-type *TP53* (log-rank test, *P* < 0.0001; Figure 3B). Further stratification analysis demonstrated that the median OS for patients with truncating *TP53* mutations and low TMB was significantly shorter compared to patients with *TP53* non-truncating mutations and low TMB (5 months vs. 8 months; log-rank test, *P* < 0.0001; Figure 3C). Multivariate Cox regression analysis adjusted for parameters such as age, sex, and ICI regimes showed that *TP53* mutations were an independent factor that was associated with shorter OS (HR = 1.41, CI = 1.05-1.89 and *P* = 0.023; Table 4) in patients with low TMB, especially those with truncating *TP53* mutations (HR = 1.40, CI = 1.13-1.73 and *P* = 0.002; Table 4).

**Table 2:** Univariate and multivariate analysis of the association between *TP53* mutations and overall survival of NSCLC patients that underwent ICI treatment.

**Variables**	**Univariate analysis**			**Multivariate analysis**		
	**HR**	**95%CI**	***P* value**	**HR**	**95%CI**	***P* value**
Age	1.01	0.74-1.38	0.948	1.04	0.91-1.19	0.584
Sex						
Female	1.00		reference	1.00		reference
Male	1.12	0.93-1.35	0.234	1.15	0.88-1.51	0.309
ICI regime						
PD-1/PD-L1	1.00		reference	1.00		reference
Combo	0.53	0.33-0.85	0.009*	0.41	0.21-0.81	0.011*
TMB (/Mb)	0.97	0.96-0.99	0.001*	0.96	0.94-0.98	0.000*
TP53						
Wildtype	1.00		reference	1.00		reference
Non-truncating	1.06	0.85-1.3	0.616	1.36	0.99-1.87	0.061
Truncating	1.36	1.05-1.76	0.019*	1.37	1.1-1.7	0.005*

NSCLC: non-small cell lung cancer; ICI: immune checkpoint inhibitors; HR: hazard ratios; CI: confidence interval; TMB: tumor mutation burden; Combo: combination of anti CTLA-4 and anti PD-1/PD-L1 therapies; /Mb: per Mega bases; * denotes P<0.05.

**Table 3:** Stratification analysis of patients with different *TP53* gene mutation status in the MSK-IMPACT NSCLC cohort.

**Variables**	**Patients with different *TP53* gene mutational status**			***P* value**
	**Wildtype**	**Non-truncating mutation**	**Truncating mutation**	
All patients	133 (%)	150 (%)	67 (%)	
Age in years				0.695
31-50	10 (7.5)	14 (9.3)	10 (14.9)	
50-60	26 (19.5)	35 (23.3)	14 (20.9)	
61-70	49 (36.8)	48 (32.0)	22 (32.8)	
>71	48 (36.1)	53 (35.3)	21 (31.3)	
Sex				0.178
Female	74 (55.6)	78 (52.0)	28 (41.8)	
Male	59 (44.4)	72 (48.0)	39 (58.2)	
ICI regime				0.060
PD-1/PD-L1	122 (91.7)	140 (93.3)	67 (100.0)	
Combo	11 (8.3)	10 (6.7)	0 (0.0)	
TMB ± SD (/Mb)	5.76 ± 4.33	12.14 ±12.09	12.92 ±10.43	<0.001*
Pathological type				0.053
Adenocarcinoma	114 (85.7)	110 (73.3)	47 (70.1)	
Squamous	11 (8.3)	24 (16.0)	10 (14.9)	
other NSCLC types	8 (6.0)	16 (10.7)	10 (14.9)	

MSK-IMPACT: Memorial Sloan Kettering Cancer Center Integrated Mutation Profiling of Actionable Cancer Targets; NSCLC: Non-cell lung cancer; ICI: immune checkpoint inhibitors; Combo: combination of anti-CTLA-4 and anti PD-1/PD-L1 therapies; TMB, tumor mutation burden; /Mb, per Mega bases; SD, standard deviation; * denotes P<0.05.

**Table 4:** The association between OS and *TP53* mutation status in low or high TMB NSCLC patient subgroups from the MSK-IMPACT cohort that underwent immunotherapy.

	**TMB < 13.8/Mb**				**TMB ≥ 13.8/Mb**			
**TP53 status**	**Cases**	**Death (%)**	**HR (95%CI)**	***P* value**	**Cases**	**Death (%)**	**HR (95%CI)**	***P* value**
2 subgroups								
wildtype	124	78 (62.90)	1.00		9	4 (44.44)	1.00	
mutation	156	111 (71.15)	1.41 (1.05-1.89)	0.023*	61	26 (42.62)	1.29 (0.44-3.28)	0.645
3 subgroups								
wildtype	124	78 (62.90)	1.00		9	4 (44.44)	1.00	
non-truncating mutations	110	74 (67.27)	1.25 (0.91-1.73)	0.168	40	16 (40.00)	1.49 (0.47-4.71)	0.500
truncating mutations	46	37 (80.43)	1.40 (1.13-1.73)	0.002*	21	10 (47.62)	1.06 (0.57-1.96)	0.855

OS: overall survival; MSK-IMPACT: Memorial Sloan Kettering Cancer Center Integrated Mutation Profiling of Actionable Cancer Targets; NSCLC: non-small cell lung cancer; HR: hazard ratios; CI: confidence interval; TMB: tumor mutation burden; /Mb: per Mega bases; * denotes P<0.05.

### *TP53* mutations were associated with significantly higher infiltration of specific immune cell subsets

**Figure 4:**
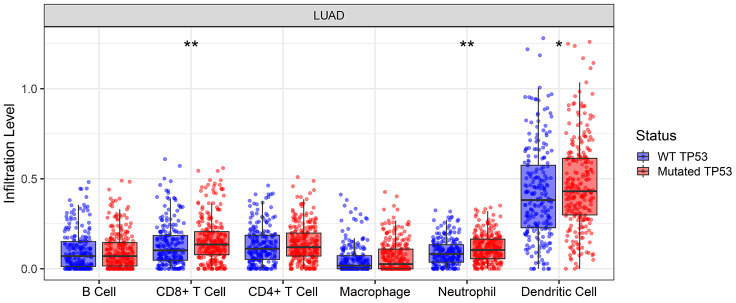
**TIMER database analysis shows the abundance of six tumor-infiltrating immune cell types in *TP53* wild-type or mutated lung adenocarcinoma tumor samples.** * denotes *P* < 0.05; ** denotes *P* < 0.01.

Tumor Immune Estimation Resource (TIMER) database analysis of lung adenocarcinoma samples showed that *TP53* mutations were associated with significantly higher infiltration of CD8+ T cells, neutrophils and dendritic cells (Figure 4).

### Prognostic model with *TP53* mutation status shows better survival prediction of NSCLC patients that underwent immunotherapy

**Figure 5:**
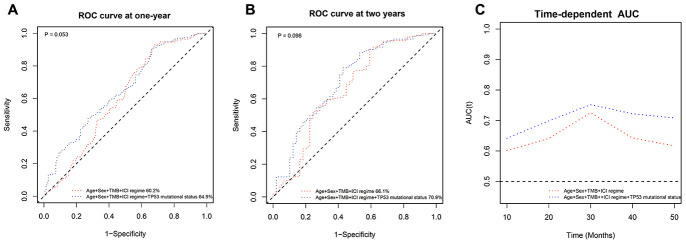
**ROC curve analysis of the prognostic prediction models with or without *TP53* mutation status.** (**A**, **B**) The ROC curves show the comparative prognostic prediction efficiency of models with age, sex, ICI regime, and TMB or age, sex, ICI regime, TMB, and *TP53* mutation status (wild-type, non-truncating or truncating mutations) as parameters at one year and two years after the 350 NSCLC patients in the MSK-IMPACT cohort received immunotherapies. (C) Time-dependent AUC of the prognostic prediction model with age, sex, ICI regime, TMB and *TP53* mutation status was higher than the time-dependent AUC of the model with age, sex, ICI regime, and TMB.

Next, we performed receiver operating characteristic (ROC) curve analysis to determine the prognostic prediction efficiency of *TP53* mutational status (wild-type, non-truncating mutations or truncating mutations). As shown in Figure 5, the area under curve (AUC) value for the prognostic model with a combination of age, sex, ICI regime, TMB and *TP53* mutational status was higher but statistically insignificant compared to the prognostic model that excluded *TP53* mutational status at one year (AUC: 64.9% vs. 60.2%; *P* = 0.052) and two years (AUC: 70.9% vs. 66.1%; *P* = 0.098) after immunotherapy (Figure 5A–5B). The time-dependent AUC of the prognostic model with *TP53* mutational status was higher than the model without *TP53* mutational status (Figure 5C).

## DISCUSSION

Previous reports show that *TP53* is an important tumor suppressor gene that determines cancer initiation, growth and progression, and is mutated in nearly 50% of all NSCLC patients [16]. Our analysis also shows that the *TP53* gene is mutated in over 50% NSCLC patients, with truncating mutations in the *TP53* gene accounting for 19.1% and 13.5% among the patients in the MSK-IMPACT- and MSK-NSCLC cohorts, respectively. A couple of studies have demonstrated that *TP53* mutations are positively associated with immunotherapeutic outcomes in NSCLC patients [17, 18], but, these results have not been validated. Our analysis shows that *TP53* mutations, particularly the *TP53* truncating mutations, are negatively associated with immunotherapeutic outcomes in NSCLC patients, especially in the low TMB subgroup. Some of non-synonymous mutations such as missense mutations result in a single amino acid change that may not dramatically change TP53 protein expression and function [19]. However, truncating mutations may cause nonsense-mediated decay of the premature mRNAs resulting in significantly lower levels of the TP53 protein as well as loss of function [12, 20, 21]. Our study shows that the OS rates are not significantly different for NSCLC patients with wild-type *TP53* and those with non-synonymous *TP53* mutations. However, the overall survival is significantly lower in NSCLC patients with truncating *TP53* mutations compared to those with wild-type *TP53*.

Previous studies suggest that tumor-infiltrating immune cells play an important role in the survival outcomes of patients treated with immunotherapy for various types of cancers [22, 23]. Our research shows that the infiltration status of three tumor-infiltrating immune cell types, CD8^+^ T cells, neutrophils, and dendritic cells, is significantly associated with the mutational status of the *TP53* gene and may impact survival outcomes of immunotherapy. Moreover, a previous study showed that the tumor-killing efficiency of cytotoxic T-lymphocytes is reduced when the cancer cells express mutant TP53 protein compared to those expressing the wildtype TP53 [24]. This suggests adverse immunotherapy outcomes for patients with mutant *TP53* tumors. The results of our study are consistent with these findings, but, rigorous large-scale studies are necessary to confirm.

The survival outcomes are significantly higher for NSCLC patients with higher TMB after receiving immune checkpoint inhibitors therapy [6, 8]. However, stratification analysis has not been performed to identify prognostic biomarkers for immunotherapy outcomes in NSCLC patients with low TMB. Moreover, the cutoff value for TMB was defined as 10 or 13.8 per mega-bases in previous studies [6, 8]. Therefore, majority of NSCLC patients were classified in the low TMB group. In our analysis, 80% of the NSCLC patients were classified in the low TMB group. Effective prognostic prediction biomarkers are required to identify NSCLC patients that are suitable to undergo immunotherapy. Our study demonstrates that *TP53* truncating mutations are a negative independent predictive biomarker for NSCLC patients. These findings demonstrate the potential of *TP53* mutations as a prognostic biomarker for NSCLC patients. However, our findings need to be validated by larger clinical trials.

## MATERIALS AND METHODS

### NSCLC patient datasets

We downloaded two independent datasets from the publicly available cBioPortal database, namely, the MSK-IMPACT dataset [25], which includes 350 NSCLC patients that received immunotherapy, and the MSK-NSCLC dataset (MSK, Cancer Cell 2018) [15], which includes 75 NSCLC patients that had received immunotherapy.

### Survival analysis of MSK-IMPACT NSCLC dataset

We divided the 350 NSCLC patients from the MSK-IMPACT dataset into 2 groups based on the nonsynonymous mutation status of the *TP53* gene, namely, *TP53* wildtype and *TP53* mutant groups. The NSCLC patients with synonymous mutations were included in the wild-type group because the mutations did not change the protein length, structure, and expression significantly. We then plotted the Kaplan-Meier survival curves and performed univariate Cox proportional hazards regression analysis of the two groups to evaluate the association between *TP53* mutation status and OS of NSCLC patients. Since truncating mutations such as frameshift insertions or deletions, nonsense, and splice-site mutations can alter the function of the proteins significantly, we then divided the NSCLC patients into 3 groups, namely, those with wild-type *TP53* (n=133)*,* truncating *TP53* mutations (n=67), and *TP53* non-truncating mutations (n=150) and performed univariate and multivariate Cox proportional hazards regression analysis with the data adjusted for age, sex, ICI regime, and TMB. TMB was defined as the total number of nonsynonymous somatic mutations per mega-base (Mb) of the genome. We calculated the TMB in the MSK-IMPACT cohort by normalizing the total number of nonsynonymous somatic mutations to the total number of mega-bases sequenced. We used the sequencing data from a 469 genes panel [6]. We performed Kaplan-Meier survival curve analysis for the *TP53* wild-type and *TP53* truncating mutation groups of NSCLC patients. We also analyzed the truncating mutation sites in the *TP53* gene to identify any hotspot mutations using R language.

### Survival analysis of the MSK-NSCLC validation dataset

Next, we analyzed another cohort of NSCLC patients that received immunotherapy, namely, the MSK-NSCLC dataset containing 75 metastatic or unresectable NSCLC patients, and compared the PFS data to validate the association between *TP53* truncating mutations and survival outcomes of NSCLC patients undergoing immunotherapy. We compared Kaplan-Meier survival curves for patients with wild-type *TP53* (n=32) and those with truncated *TP53* mutations (n=10) using univariate Cox proportional hazards regression analysis. We also compared the PFS between the two groups of NSCLC patients.

### Stratification analysis

Next, we performed correlation analysis of the MSK-IMPACT dataset to determine the relationship between *TP53* mutations and clinicopathological variables. We observed significant correlation between *TP53* mutations and TMB status of NSCLC patients. Hence, we performed stratification analysis of OS and *TP53* status in low or high TMB subgroups. We determined the cutoff value for classifying patients into low or high TMB groups as 13.8/Mb, based on previously published data using the same dataset [6]. We then performed Kaplan-Meier survival curve analysis including the log-rank test and multivariate Cox regression analysis adjusted by age, sex, and ICI regimes for these subgroups.

### Tumor-infiltrating immune cell analysis

We used the TIMER database (https://cistrome.shinyapps.io/timer/) to determine the abundance of six tumor-infiltrating immune subsets, namely, B cells, CD4^+^ T cells, CD8^+^ T cells, neutrophils, macrophages, and dendritic cells in NSCLC tumor samples from patients with the wild-type TP53 and those with TP53 mutations. The results were derived from the sequencing data obtained from 10897 tumor samples belonging to 32 cancer types at The Cancer Genome Atlas (TCGA) database [26]. Specifically, we analyzed six subsets of tumor infiltrating immune cells in 544 TCGA lung adenocarcinoma samples using TIMER online tools.

### ROC curve analysis of the prognostic prediction models with or without *TP53* mutation status

We investigated the prognostic prediction efficiency of the *TP53* mutation status by constructing two prognostic models: (1) age, sex, ICI regime, and TMB; (2) age, sex, ICI regime, TMB and TP53 mutation status. Then, we generated ROC curves based on the one-year and 2-year survival data and also evaluated the time-dependent dynamic AUC of the two models.

### Statistical analysis

All the statistical analysis was performed using R language (version 3.5.1). The Cox proportional hazards regression analysis was used to perform univariate and multivariate analysis of the clinical variables. Stratification analysis was used to estimate the true association between clinical variables and survival outcomes by analyzing the variables in subgroups. The groups were compared using two-sided t-test and P< 0.05 was considered statistically significant.

## Supplementary Material

Supplementary Figure 1

Supplementary Table 1
