# Supplementary material for: TP53 somatic mutations are associated with poor survival in non-small cell lung cancer patients who undergo immunotherapy
Source: Aging (Albany NY). 2020 Jul 22;12(14):14556–68. doi: 10.18632/aging.103502 (PMC7425464; doi:10.18632/aging.103502)
Supplement: Supplementary Figure 1 [file aging-12-103502-s002..pdf]

SUPPLEMENTARY FIGURE

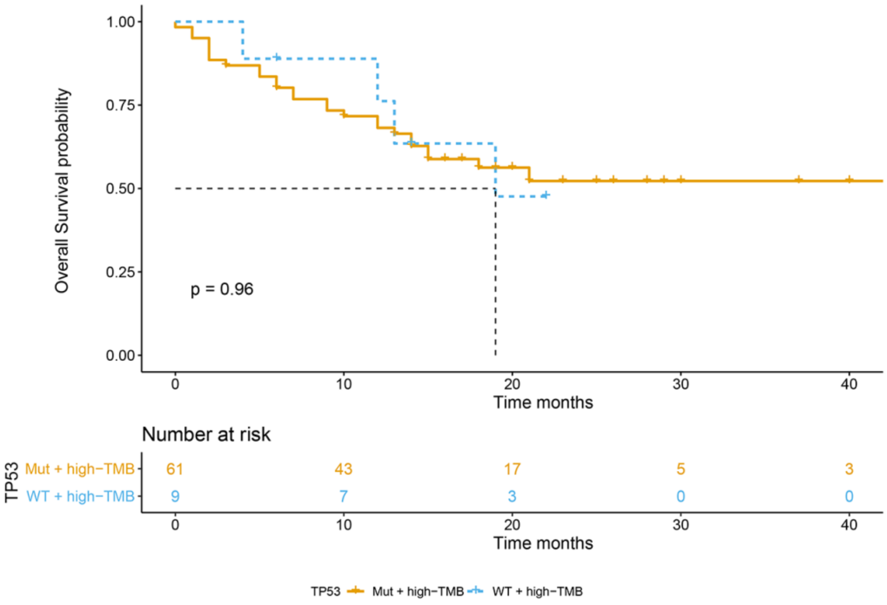

Supplementary Figure 1. Overall survival analysis shows no difference between the high TMB NSCLC patients with or without TP53 mutations from MSK-IMPACK cohort.
